# Localized Quantum Chemistry on Quantum Computers

**DOI:** 10.1021/acs.jctc.2c00388

**Published:** 2022-11-08

**Authors:** Matthew Otten, Matthew R. Hermes, Riddhish Pandharkar, Yuri Alexeev, Stephen K. Gray, Laura Gagliardi

**Affiliations:** † HRL Laboratories, LLC, 3011 Malibu Canyon Road, Malibu, California90265, United States; ‡ Department of Chemistry, Pritzker School of Molecular Engineering, James Franck Institute, Chicago Center for Theoretical Chemistry, University of Chicago, Chicago, Illinois60637, United States; § Computational Science Division, Argonne National Laboratory, Lemont, Illinois60439, United States; ∥ Center for Nanoscale Materials, Argonne National Laboratory, Lemont, Illinois60439, United States; ⊥ Argonne National Laboratory, Lemont, Illinois60439, United States

## Abstract

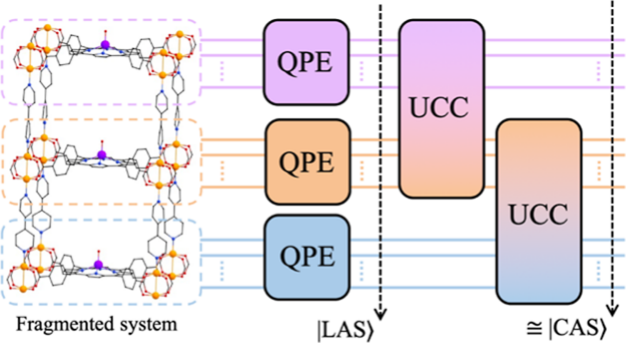

Quantum chemistry calculations of large, strongly correlated systems are typically limited by the computation cost that scales exponentially with the size of the system. Quantum algorithms, designed specifically for quantum computers, can alleviate this, but the resources required are still too large for today’s quantum devices. Here, we present a quantum algorithm that combines a localization of multireference wave functions of chemical systems with quantum phase estimation (QPE) and variational unitary coupled cluster singles and doubles (UCCSD) to compute their ground-state energy. Our algorithm, termed “local active space unitary coupled cluster” (LAS-UCC), scales linearly with the system size for certain geometries, providing a polynomial reduction in the total number of gates compared with QPE, while providing accuracy above that of the variational quantum eigensolver using the UCCSD ansatz and also above that of the classical local active space self-consistent field. The accuracy of LAS-UCC is demonstrated by dissociating (H_2_)_2_ into two H_2_ molecules and by breaking the two double bonds in *trans*-butadiene, and resource estimates are provided for linear chains of up to 20 H_2_ molecules.

## Introduction

Chemical systems with many close-lying electronic states or, more generally, strongly correlated electrons pose a significant challenge for modern electronic structure theories in computational quantum chemistry.^1−5^ When transition metals or heavier elements with partially filled valence d/f orbitals are involved, degenerate and nearly degenerate electronic states are common, and single-reference electronic structure methods, such as the Kohn–Sham density functional theory often fail.^6−10^ In these situations, one has to use multireference methods to generate multiconfigurational wave functions and accurately describe these near degeneracies.^9,11,12^

Scientists also want to compute properties of large chemical systems or solids with accurate quantum chemistry methods, in spite of steep computational requirements. One way to achieve such computations is to use fragmentation methods. Many variations of fragmentation methods exist,^13−16^ but the common feature is that a large molecular system is divided into fragments and quantum-mechanical calculations are performed on the fragments. An especially important case is the application of fragmentation methods to multireference wave functions because of the exponential explosion of the computational cost with respect to the size of the active space of electronic configurations.

In the complete active space self-consistent field (CASSCF) method,^17^ all the electronic configurations that can be formed for a given number of active electrons distributed in a given number of active orbitals are included in the wave function. Thus, the wave function scales exponentially with the number of active electrons and orbitals, and the method has only limited application to chemically relevant systems. If one wants to study systems containing, for example, several transition metals,^18−22^ the active site of a protein,^23^ or extended organic chains in their ground and excited states,^23,24^ more affordable multireference methods have to be developed. This is one of the major challenges of modern electronic structure theory.

Reducing the computational cost of CASSCF or other multiconfiguration self-consistent field calculations is pursued both in the development of new well-motivated theoretical approximations and in the application of new developments in computational hardware.^25,26^ On the theoretical side, one strategy is to identify subspaces of the CAS that can be treated on different footings^27,28^ or interact with one another only weakly.^29−33^ The localized active-space self-consistent field (LASSCF) method,^34−37^ also known as the cluster mean-field (cMF) method,^38^ is an example of such a strategy. LASSCF is designed for applications in which electrons are strongly correlated in different weakly interacting physical regions of a molecule and approximates the strongly correlated part of the wave function as a single antisymmetrized product of subspace wave functions. The computational cost of LASSCF is a linear function of the number of such unentangled subspaces.

Some of the authors have recently shown that LASSCF accurately reproduces the CASSCF spin-state energy gaps of bimetallic compounds and the simultaneous dissociation of two double bonds in bisdiazene at a significantly reduced cost.^35,36^ However, LASSCF fails to recover any electron correlation between fragments, for example, in the *cis*–*trans* isomerization of stilbene and similar systems.^37^ Moreover, methods to restore the missing correlation variationally,^39^ perturbatively,^38,40^ or via the coupled-cluster (CC) approach^41^ on classical computers must usually enumerate a general many-body basis for each fragment. That is, they inherit the complications of multireference perturbation and CC theories^9,42^ over traditional single-reference perturbative or truncated CC corrections based on second quantization.^43,44^

Recently, the development of quantum computers has led to an increased interest in novel quantum algorithms, especially for computational quantum chemistry, which is widely seen as a potential “killer app” of quantum computers.^45−47^ The quantum phase estimation (QPE) quantum algorithm^48^ can potentially offer exponential speedups when large fault-tolerant quantum computers are available,^49,50^ under the assumption that an initial state with non-negligible overlaps can be prepared.^51,52^ Additionally, the variational unitary coupled cluster (UCC) requires only a polynomial number of gates to represent on a quantum computer, whereas representing the same ansatz classically has no known polynomial solution.^53,54^ For the noisy, intermediate-scale quantum (NISQ)^55^ devices that we have today, these algorithms are not tenable because they require coherence times far beyond what is available. Variational algorithms, such as the variational quantum eigensolver (VQE),^54^ have been used to perform calculations of the ground-state energy of small molecules, with limited accuracy, on NISQ devices.^56−58^ Quantum algorithms that have less stringent requirements compared with full QPE and at the same time, accuracy beyond that demonstrated by variational algorithms, such as VQE, will be required to productively use the progressively larger and higher quality quantum devices as they become available in the next few years.

In this paper, we describe a framework for such quantum algorithms, inspired by classical LASSCF. The wave function within a fragment is solved by using one method (e.g., QPE), and correlation between fragments is encoded variationally by using an ansatz that entangles the fragments. This approach goes beyond what can be achieved with classical fragment methods, such as LASSCF, by providing additional correlation between fragments, while significantly reducing the total computational time (estimated via the number of gates) compared with full QPE.

## Theory

### Multireference Methods with Exponential Scaling

We seek to find the ground state of the second-quantized molecular Hamiltonian for a given number of *M* electrons
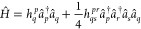
1where  creates (annihilates) an electron in spin-orbital *p*; *h*_*q*_^*p*^ and *h*_*qs*_^*pr*^ are the one- and antisymmetrized two-electron Hamiltonian matrix elements, respectively; and repeated internal indices are summed. Generally, for *N* spin-orbitals, *Ĥ* has a sparse-matrix representation in a space of size  and has *O*(*N*^4^) elements. Full-configuration interaction (FCI) determines the exact energy within a given one-electron basis set (the FCI energy) at exponential cost. Methods, such as CASSCF (and its restricted^27,28^ and generalized^29,59^ active space approximations) or selected configuration interaction (CI),^60,61^ can go beyond FCI in the system size, maintaining comparable accuracy, but still scale exponentially. The density matrix renormalization group (DMRG)^62−65^ and coupled cluster methods^44^ can scale polynomially but introduce (sometimes uncontrollable) approximation errors. Here, we briefly describe the LASSCF algorithm,^34,36^ which will serve as the basis for our fragment-based quantum algorithms.

### LASSCF

In LASSCF, the wave function of a molecule is approximated as
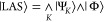
2where |Ψ_*K*_⟩ is a general many-body wave function describing *M*_*K*_ electrons occupying *N*_*K*_ active orbitals of the *K*th “fragment” or “active subspace,” |Φ⟩ is a single determinant spanning the complement of the complete active space, and the wedge operator (“∧”) implies an antisymmetrized product.

In the variational^36^ implementation of LASSCF, this wave function is obtained by minimizing the LAS energy
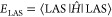
3with respect to all orbital rotations and CI vectors defining |LAS⟩. This is accomplished by introducing a unitary operator (see the Supporting Information of ref (36)) that is parameterized in terms of all nonredundant transformations of the orbitals and CI vectors
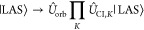
4where
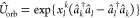
5
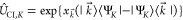
6where *k*, *l* index individual spin-orbitals in two different subspaces (including the inactive and virtual subspaces outside of the CAS) and where  is a determinant or configuration state function. First and second derivatives of eq 3 with respect to the generator amplitudes (*x*_*l*_^*k*^ and ) are obtained by using the Baker–Campbell–Hausdorff (BCH) expansion, and the energy is minimized by repeated applications of the preconditioned conjugate gradient (PCG) method.^66,67^

The orbital unitary operator, , corresponds to the UCC correlator truncated after the first (“singles”) term
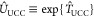
7
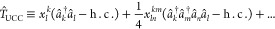
8

The use of the more general cluster operator, eq 8, in place of the orbital rotation unitary operator, eq 5, corresponds to a multireference UCC method^68^ built on top of a |LAS⟩ reference wave function. Such a method is expected to be more flexible than LASSCF itself, in that doubles and higher order cluster amplitudes could encode electron correlation and entanglement between active subspaces. This would require the reference wave function, |LAS⟩, to be updated by explicit exponentiation of the general cluster operator, eq 8, after each execution of the PCG algorithm. On classical computer hardware, however, this is not an efficient way to extend LASSCF. The LAS wave function is equivalent to other methods that also express the wave function in the product of localized states—namely, the active space decomposition (ASD),^31,69−71^ rank-one basis,^32,72^ and cMF.^38^ Classical approaches to couple the various fragments that use the basis of these tensor product states to obtain molecular wave functions have been proposed. The ASD-DMRG algorithm,^71^ cluster many-body expansion,^73^ and tensor product selected configuration interaction approach^74^ have shown to be effective in restoring the coupling between the fragments.

### LAS Methods on Quantum Computers

Here, we describe an algorithm for molecular calculations that goes beyond the limited accuracy of standard VQE,^57,58^ while having dramatically reduced computational complexity compared with QPE [see the Methods section]. The algorithm exploits the structure of the molecule by separating it into coupled fragments, as is done in the classical algorithm, LASSCF. The quantum algorithm, however, goes beyond classical LASSCF by providing some degree of entanglement between the fragments.

The algorithm begins by segmenting the orbital active space of a given molecule into distinct fragments defined by non-overlapping orbital subspaces, as in classical LASSCF. For instance, orthogonalized atomic orbitals (AOs) generated by using the meta-Löwdin method^75^ can be sorted into localized fragments and then projected onto a guess for the CAS of a given molecule to produce localized active orbitals. We construct an effective Hamiltonian that omits non-mean-field interfragment interactions, resulting in a sum of local fragment Hamiltonians
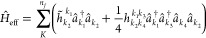
9where *k*_1_, *k*_2_, ... index distinct active orbitals of the *K*th fragment and where
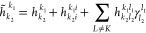
10where *i* and *l*_*n*_ index, respectively, inactive orbitals [i.e., those defining |Φ⟩ in eq 2] and active orbitals of the *L*th fragment and where  is a one-electron reduced density matrix element for spin-orbitals *l*_1_ and *l*_2_
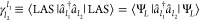
11

Given a set of localized active orbitals that minimize the LASSCF energy, if the density matrices in eq 10 are obtained from a classical LASSCF calculation on the same system, then the QPE algorithm applied to  generates the active-space part of the LASSCF wave function, |QLAS⟩ = ∧_*K*_|Ψ_*K*_⟩, on the quantum computer. The same result is achieved if density matrices are obtained self-consistently from the QPE evaluation. If the density matrices are obtained in some other way, for instance from |HF⟩, then an approximation to the LASSCF wave function is obtained.

The QPE step provides the initial |QLAS⟩ for each fragment step by repeating the measurement of the phase until it is consistent with the phase representing the ground-state energy, which collapses the system into the ground-state wavefunction. This introduces some overhead, as each fragment will need to be in the ground state to continue to the next step. Furthermore, a full QPE solve, estimating the ground-state energy, must be performed initially to provide a comparison value.

A sequence of UCC with single and double (UCCSD) circuits, with variable parameters, is then applied across *m* fragments each (which we term *m*-local), leading to the LAS-UCC wave function
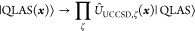
12where  is the UCCSD ansatz, including only creation/annihilation operators within the *m* fragments that it spans, ζ is a list of fragment indices of size *m*, and ***x*** are the associated single and double cluster amplitudes. The factorization of eq 8 implied by eq 12 is based on the intuition that physically adjacent active subspaces are likely to be more strongly entangled to one another than subspaces on opposite ends of a large molecule. The parameters of the UCCSD circuit are varied to minimize the total energy of the full system, as in the VQE [see also the Methods section]
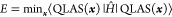
13

**Figure 1:**
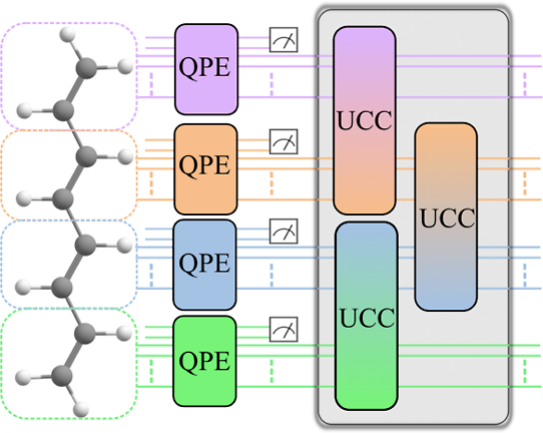
Diagram of example circuit using LAS-UCC. The system of interest is first separated into distinct fragments. QPE (using necessary ancilla qubits) is used on each fragment to solve for the approximate unentangled ground state. Correlation between fragments is then added in, variationally, through a UCC ansatz.

A schematic representation of the described circuit is shown in Figure 1. This provides an electron correlation between the fragments, in a way that scales exponentially on classical computers, but only polynomially on quantum computers. Moreover, this procedure provides a better estimate of the ground-state energy than the product wave function or the UCCSD would provide alone. Like LASSCF, this energy is an upper bound to the FCI ground-state energy but unlike LASSCF, this method is nevertheless not strictly variational (despite the use of VQE) because the initial product-state wave function, ∧_*K*_|Ψ_*K*_⟩, is not variationally reoptimized in the presence of the UCCSD correlators (in other words, |QLAS⟩ does not satisfy the Hellmann–Feynman theorem^76−78^). The QPE circuits could also be replaced with a local variational ansatz, leading to a fully variational algorithm, which we term LAS-VQE and describe in the Supporting Information.

To summarize, estimating the LAS-UCC energy for a specific set of variational parameters requires the following steps: (i) prepare the initial state, via localized QPE circuits; (ii) apply the *m*-local UCCSD ansatz to the prepared initial state; and (iii) measure the state of the qubits. This is repeated many times for a given set of parameters to obtain an estimation of a single observable. As is standard in all VQE algorithms, this is repeated for many observables, which are then summed on the classical device to obtain the estimate of the energy.^54^ A classical optimizer controls the values of the parameters, and the whole estimation must be repeated for each set of parameters until convergence. The two differences between a standard VQE with a full UCCSD and LAS-UCC are the different initial state and the reduced *m*-local ansatz. The two primary differences between the standard QPE and LAS-UCC are the reduced, local QPE circuits, and the addition of the variational *m*-local UCCSD circuit.

To understand the large improvement in computational complexity of our approach, we focus on a system of *n*_*f*_ fragments, with the number of orbitals per fragment, *N*_*K*_, constant as the number of fragments grows. The total system size is defined by *N* = *N*_*K*_*n*_*f*_ orbitals. We also assume that each fragment interacts with only the *m* geometrically nearest fragments and that *m* does not grow with *n*_*f*_. These are reasonable assumptions for many interesting molecules and mirror the assumptions made in classical LASSCF. Under these assumptions, the QPE solver for the unentangled fragments does not grow with *N* because *N*_*K*_ is assumed to be fixed while *n*_*f*_ grows. The number of small QPE sections grows linearly with the number of fragments, of course. Typically, the Jordan–Wigner transformation would introduce an *O*(*N*) term to enforce anticommutation relations among the orbital creation and annhilation operators. However, in the case of linear chains, as we study here, ordering the orbitals such that all up and down occupied and virtual orbitals in a given fragment are close, the high-weight *Z* part of the Jordan–Wigner transformation effectively cancels out, causing no scaling with the total number of orbitals. See the Supporting Information for more details. Together, this leads to an overall  (linear) number of gates to solve for the *n*_*f*_ unentangled product wave functions. The UCCSD correlator, which is then applied, has  terms in the cluster operator for each correlator because the UCCSD circuit spans only *m* fragments. Neither *m* nor *N*_*K*_ grows with the total size (number of spin-orbitals) of the system, *N*. The number of *m*-local correlators grows as *O*(*n*_*f*_). Again, by careful ordering of the orbitals, the Jordan–Wigner transformation does not introduce any scaling overhead. The complexity of the *m*-local UCCSD correlator is then  (linear). This creates an overall linear scaling in the number of gates for linear chain geometries, with respect to only the total size of the system, *N*, and is polynomially (*O*(*N*^4^)) better than performing QPE alone, while providing accuracy above VQE using the UCCSD ansatz and classical LASSCF. Many of the gates can be done in parallel, such as the local QPE circuits and the different *m*-local UCCSD correlators, leading to an expected overall sub-linear depth. If the fragments are coupled in a geometry more complicated than a linear chain, the UCCSD correlator will potentially incur the *O*(*N*) Jordan–Wigner overhead, leading to an overall *O*(*N*^2^) scaling for arbitrary geometries with an expected *O*(*N*) depth.

### Illustrative Molecular Systems

**Figure 2:**
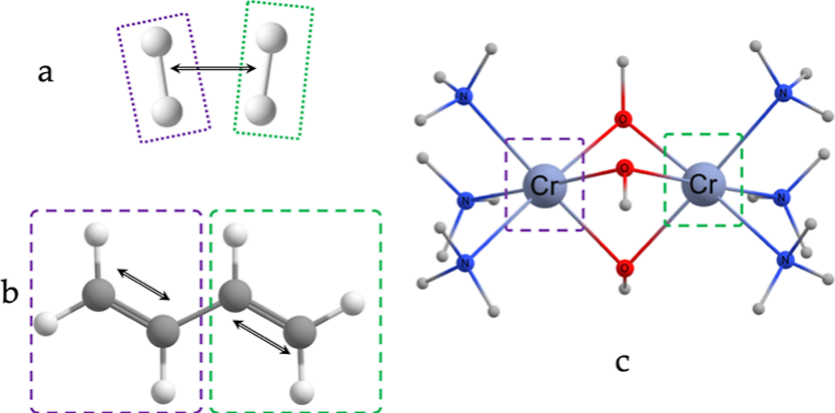
Three model systems used for testing. (a) Asymmetric hydrogen dimer, . Each H_2_ molecule is a fragment described by a 2-electron, 2-spatial orbital, or (2,2) active subspace in the dimer’s LAS wave function. The potential energy surface is scanned along the distance between the two H_2_ bond midpoints, indicated by the black double line. (b) *trans*-Butadiene molecule at its CASSCF(8,8)/6-31G ground-state equilibrium geometry. Dashed boxes depict the two notional fragments containing the two (4,4) active subspaces in the LAS wave function. Black double lines indicate the internal coordinate along which the potential energy surface is scanned; the two terminal methylene units are simultaneously removed from the central acetylene unit. (c) [Cr_2_(OH)_3_(NH_3_)_6_]^3+^ molecule—the structure is obtained by truncating the ligands of the molecule in reference 79 at the nitrogen atoms and capping them with hydrogen atoms to maintain the charge.

In the calculations discussed below, we consider three systems, as depicted in Figure 2. The first, shown in Figure 2a, is a simplistic model of weakly interacting fragments, consisting of two H_2_ molecules at various distances between their two midpoints using a minimal STO-3G AO basis set, and the two active subspaces in the LAS wave function correspond to the active spaces of the two H_2_ molecules. We use this small basis set because of the size limitations of today’s quantum computers and simulations. The bond lengths and internal angles of this system are set arbitrarily to remove point group symmetry so that differences between various methods are not obscured by the simplicity of a symmetrized electronic wave function. The interaction between the two fragments in this model system are weak, and the LAS wave function is, therefore, expected to provide an excellent model of the FCI wave function except when the distance between the two molecules is very small. We additionally extend this system up to 20 H_2_ in a linear chain, where we estimate only the total number of quantum resources necessary.

The second system, as depicted in Figure 2b, is the *trans*-butadiene molecule. The potential energy surface of this molecule is scanned along the internal coordinate corresponding to the simultaneous stretching of both the C=C double bonds, leading to the removal of two methylene units from a central C_2_H_2_ (distorted acetylene-like) unit. In the LAS wave function, the molecule is divided into two fragments split across the central C–C bond, and each fragment is described by a (4,4) active subspace. Several molecular orbitals are, therefore, left inactive, described by an unfragmented single determinant. We employed the 6-31G AO basis set in this case.

The *trans*-butadiene system is a chemical model of the case of two strongly interacting units in a system, where the value of the stretching internal coordinate is a proxy for the strength of electron correlation. Near the equilibrium geometry, dividing the active space into two fragments is chemically reasonable: each fragment encloses one π-bond, and inasmuch as electron correlation affects the system at all, it is a reasonable approximation to consider it only locally. However, as the C=C double bonds are elongated, electrons from the two broken π bonds recouple across the central C_2_H_2_ unit, which spans the fissure between the two LAS fragments. The LAS wave function cannot model a π bond in this position, and the LASSCF method breaks down. The active orbitals from the CASSCF and LASSCF calculations at the equilibrium geometry and the dissociated geometry are shown in Figures S6 and S7 in the Supporting Information.

The molecule depicted in Figure 2c is a model for a tris-(μ-hydroxo)-bridged chromium compound studied experimentally and theoretically.^79−83^ Accurately modeling the spin-coupling in bimetallic compounds is important for many applications including molecular-magnet and qubit design. Compounds with more than one metal center lend themselves to a very intuitive fragmentation scheme of active subspaces localized on each center. The unpaired electrons in the partially filled d orbitals of such compounds are typically localized in space allowing us to model these metals as interacting individual spin centers with a physically meaningful “local” S value equal to the half of the number of unpaired electrons localized on each metal center. With multiple such metals, the locally high-spin centers can couple with each other either in a ferromagnetic [high spin (HS)] or anti-ferromagnetic [low spin (LS)] arrangement. The nature of the coupling between the centers dictates the orientation of the spin in the ground state. The commonly used Heisenberg–Dirac–Van Vleck model^84−86^ uses the *J* coupling parameter to describe the coupling between the two centers. The sign of *J* indicates the orientation (negative for antiferromagnetic and positive for ferromagnetic) and the magnitude reflects the strength of the coupling. The *J* value can vary significantly for different metals and linkers and based on the spatial symmetry, chemical environment, temperature, and so forth. Typically, this coupling constant is relatively small (especially if the metal centers are separated by linkers) between 0.1 and 100 cm^–1^. This means that the mean-field interaction between the spin centers (that LASSCF includes) is not sufficient to make accurate predictions. Moreover, mechanisms like direct exchange and super exchange are conceptually two-body phenomena that are ignored in a LASSCF wave function. Thus, a method that accounts for an explicit correlation between the two fragments beyond LASSCF is necessary for such systems. In this work, the aliphatic ligands were truncated at the nitrogen atoms and capped with hydrogen atoms to balance the charge. An active space of 6 unpaired electrons in the 6 singly occupied 3d orbitals is considered. The def2-svp basis was used for the C, N, O, and H atoms, while the def2-tzvp basis was used for the Cr atoms.

## Results and Discussion

### LAS-UCC

We demonstrate the efficacy of our framework by simulating the three benchmark molecules, (H_2_)_2_, *trans*-butadiene, and the chromium compound described above. We compare three methods: LASSCF, CAS CI in the basis of LASSCF orbitals (CASCI), and our new algorithm, LAS-UCC. LASSCF represents the best unentangled set of wave functions and is equivalent to the solution after the QPE circuits but before the use of the UCCSD ansatz. Note that CASCI is slightly different from CASSCF because the orbitals are not variationally reoptimized. CASCI solves for the FCI wave function within the active space; in this case, it is equivalent to using QPE across the whole molecule and represents the reference result in these studies. Figure S5 in the Supporting Information shows that the CASCI with these orbitals is a good approximation to the corresponding CASSCF (8,8), which is at most only 17.7 mHartree lower in energy than CASCI.

**Figure 3:**
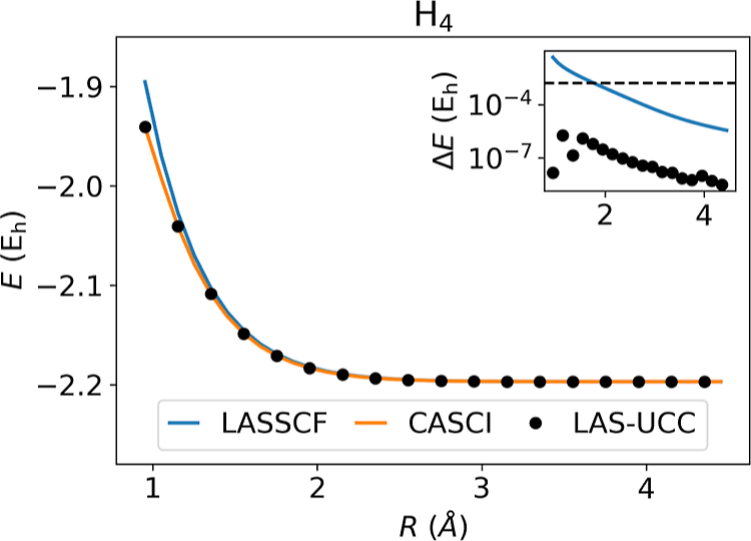
Energies for (H_2_)_2_ calculated by CASCI, LASSCF, and LAS-UCC. The inset shows the error, with respect to CASCI, of LASSCF and LAS-UCC. The black dashed line represents chemical accuracy. LAS-UCC is able to obtain chemical accuracy, with respect to CASCI, at all distances. LASSCF cannot obtain chemical accuracy at sufficiently short distances.

Figure 3 shows the results with the different methods for the hydrogen dimer as the two H_2_ molecules are pulled apart. We see that LASSCF, CASCI, and LAS-UCC agree except for very small distances where LASSCF no longer provides accurate energies.

**Figure 4:**
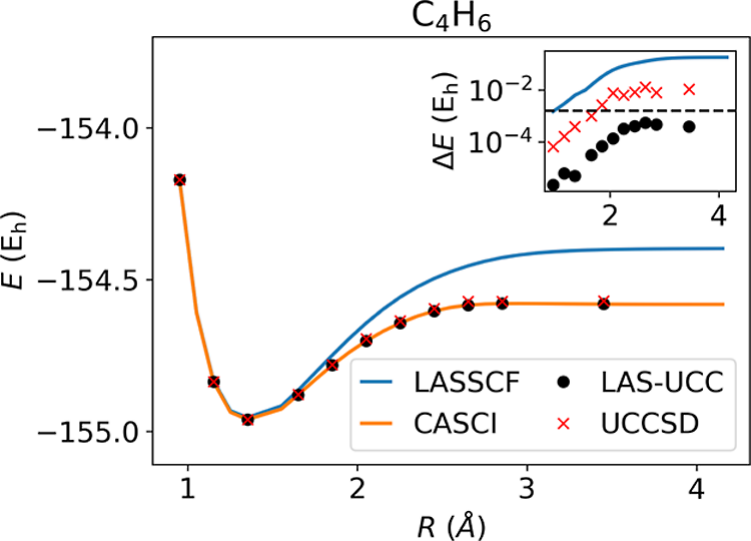
Energies for C_4_H_6_ calculated by CASCI, LASSCF, and LAS-UCC. The inset shows the error, with respect to CASCI, of LASSCF and LAS-UCC. The black dashed line represents chemical accuracy. LAS-UCC obtains chemical accuracy across the potential energy surface, whereas LASSCF, which cannot accurately represent the correlation between the fragments, fails to obtain chemical accuracy for most points.

Figure 4 shows the results for *trans*-butadiene, a model of strongly correlated fragments. Here, as the terminal methylene units are removed, the interfragment correlation grows as a double bond is formed between the fragments. The UCCSD ansatz can accurately represent this level of entanglement, allowing LAS-UCC to achieve nearly CASCI accuracy, whereas LASSCF fails to account for this entanglement. With a standard Hartree–Fock initial state, as is typically done in VQE, the UCCSD ansatz is unable to obtain chemical accuracy for the large distances. We also attempted to use the so-called “hardware-efficient” ansatz^58^ but were unable to obtain results significantly better than Hartree–Fock using depths up to 10 (which corresponds to a similar number of parameters as the UCCSD ansatz) at equilibrium.

Motivated by the success of LAS-UCC for the *trans*-butadiene test case, we used the UCC correlator to recouple the localized subspaces in a bimetallic compound in Figure 2c. The two chromium(III) ions have three unpaired electrons each that exhibit strong anti-ferromagnetic coupling according to experimental observations. We chose to model this with a minimal active space of 6 electrons in 6 orbitals—which include the three singly-occupied d orbitals on each Cr ion. As shown in previous theoretical studies, one needs a much larger active space along with post-MCSCF methods to accurately predict the experimentally observed value of *J*. While we cannot thus expect the (6,6) calculation to be accurate, it serves as an excellent example for how methods like LAS-UCC can be used once we have the resources to study large active spaces. Table 1 shows the energies for the HS (in this case, a septet) and the LS (in this case, a singlet) states along with the *J* value computed from these energies using the Yamaguchi formula. The CASSCF results give a value of −13.8 cm^–1^ indicating a reasonably strong anti-ferromagnetic coupling (as the experiment would suggest). The LASSCF calculations, however, give a *J* of +11.2 cm^–1^, which is qualitatively inaccurate. The CASCI calculations with the LASSCF orbitals give a *J* of −11.6 cm^–1^ indicating the dominant problem with the LASSCF is the lack of correlation rather than the shape of the orbitals. The LAS-UCC calculations agree with the CASCI and thus predict the qualitative behavior (that of a LS ground state) accurately.

**Table 1:** Absolute Energies (hartree), Spin Gaps, and *J* Values for System **c** Computed Using Various Methods

method	HS energy	LS energy	HS–LS gap (cm^–1^)	*J* (cm^–1^)
CASSCF	–2649.1455510	–2649.1463079	–166.1	–13.8
LASSCF	–2649.1455510	–2649.1449395	134.2	11.2
CASCI@LAS orbitals	–2649.1455510	–2649.1461837	–138.9	–11.6
LAS-UCCSD	–2649.1455510	–2649.1461837	–138.9	–11.6

## Resource Estimates

To demonstrate the scaling advantage of our method, we perform resource estimation for the number of logical quantum gates necessary for several different quantum algorithms: the QPE algorithm over the full unfragmented molecule; the UCCSD ansatz over the full unfragmented molecule; and the two steps of our proposed LAS-UCC method, the fragmented QPE and the 2-local UCCSD (which corresponds to the circuit, as depicted in Figure 1). We estimate the number of resources needed for the QPE algorithm if only a single Trotter time step were needed; *O*(1000) time steps will be needed for typical systems to get to chemical accuracy.^47,87^ Note that these estimates represent only the number of two-qubit CNOT gates, which we use as a primary gauge of the number of total resources. Single-qubit gates are also necessary; the estimates for these resources can be found in the Supporting Information and scale similarly to the number of CNOT gates. We also note here that we are only comparing the scaling number of gates; QPE, with a sufficiently good initial state and enough Trotter states, will of course be the most accurate of all compared algorithms.

**Figure 5:**
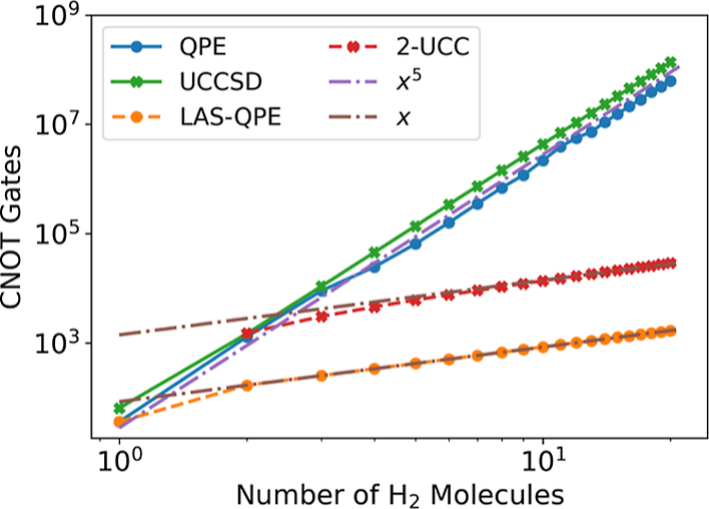
Estimated two-qubit gate counts using various algorithms. The QPE estimates assume only a single Trotter step; *O*(1000) will need to be taken to obtain chemical accuracy. Polynomials of various orders have been plotted to demonstrate the scaling. Our algorithm, LAS-UCC, requires both the LAS-QPE and 2-UCC circuits and thus has an overall *O*(*N*) scaling, compared with the *O*(*N*^5^) scaling of UCC and QPE.

We use a model system of an increasing number of H_2_ molecules and look at how the number of CNOT gates increases as the number of molecules increases, as shown in Figure 5. As the number of H_2_ molecules increases, the number of gates needed for all methods also increases. As predicted in the complexity analysis of QPE [see the Methods section], the total number of gates for a single Trotter step in the QPE algorithm grows as *O*(*N*^5^). Similarly, the number of gates needed for a global UCCSD ansatz also grows as *O*(*N*^5^), as expected.^57^ This result is compared with the much smaller number of gates necessary to implement the two steps of our LAS-UCC algorithm. As expected, both the QPE and UCCSD parts of LAS-UCC provide dramatic scaling advantages, with the 2-local UCCSD ansatz and the QPE of the reduced Hamiltonian both scaling as only *O*(*N*). We note that, in addition to evaluating the quantum circuits here, an additional optimization loop is needed when using the UCCSD ansatz, whether it is global or 2-local. Using a 2-local UCCSD ansatz also greatly reduces the number of parameters that need to be optimized compared with a global UCCSD ansatz.

The number of gates needed to perform a single Trotter step comprises a majority of the scaling of the final algorithm. More precise resource estimates would include overheads from the following items: (i) the number of necessary ancilla qubits, which would affect the final precision^88^ and can be roughly estimated to be 10 or less;^50^ (ii) the number of Trotter steps needed, which depends on the desired accuracy and specific product formula used;^89^ (iii) the quantum error correcting code used;^90^ and (iv) the specifications of the exact quantum hardware being used. Each QPE circuit would need to be repeated many times, both to obtain the desired precision on the sampling statistics and because the initial state’s overlap with the true ground state is typically less than one.^91^ During the optimization of the variational parameters of the UCCSD ansatz, the number of iterations needed depends on the specific optimization algorithm used and the variational landscape of UCCSD.^92^ Estimates including detailed analysis of many of these factors show that the run times for a full QPE algorithm can range on the order of hours to years.^93−95^ Because these estimates are so dependent on many underlying assumptions, we instead focus on just the core component of the more detailed estimates: one Trotter step. When the resources for this important subroutine are lowered, the overall time for QPE will likely be lowered by a similar amount. The optimization loop will add additional overhead, of course, but we expect this factor to be much smaller than the sizable savings in the QPE subroutine.

## Discussion

Here, we compare LAS-UCC with the two quantum algorithms that it is composed of the following: QPE and variational UCCSD. Compared with global QPE, LAS-UCC reduces the total quantum resource cost by approximating the system with noninteracting fragments and adding in some interaction between fragments (those described by a UCCSD ansatz spanning the fragments). This in general reduces the accuracy; but as shown in the preceding sections, LAS-UCC provides accuracy comparable to CASCI (and therefore, global QPE) for the systems considered here. The *trans*-butadiene molecule is a model for larger, more complicated systems of strongly interacting units. Many single molecular magnets have such pockets of strong correlation localized on the metal centers, which moderately interact with each other.^96,97^ With LAS-UCC, we not only can obtain the wave function efficiently but also can selectively couple the fragments with the UCC correlator, offering further insights into the nature of these interactions. Affordable and accurate modeling of phenomena, such as singlet fission,^98,99^ in molecular crystals of conjugated organic compounds can be performed with LAS-UCC, as fault-tolerant quantum computers become available. This approach will also be used to study chemical processes involving interfragment bond formation and breaking while still treating all points on a potential energy surface at comparable footing. Though we do not perform detailed resource estimation beyond a single Trotter step or for various other overheads (e.g., number of shots, number of iterations in the optimizer, and so on), we can qualitatively contrast the other overheads for global QPE and LAS-UCC. LAS-UCC decreases the gate depth, as shown above, but adds an additional classical optimization loop, requiring evaluation of the circuits at various values of parameters along the trajectory of the optimizer. Furthermore, there will be a large increase in the number of measurements needed compared with the standard QPE, due to the need to measure each element of the Hamiltonian separately in VQE-like algorithms. Millions of shots can be required over the optimization trajectory for small molecules, even with shot-adaptive algorithms.^92^ Some of the shot overhead can be ameliorated using multiple quantum computers running in parallel because each shot is independent.

Both the global QPE and the local QPE part of LAS-UCC will have overheads due to the initial state’s (before the QPE circuits) overlap.^91^ Though there are no formal proofs, numerical evidence suggests that this overlap decreases with increasing size of the molecular system.^100,101^ Given that the local QPE solvers in LAS-UCC are substantially smaller, it is possible that LAS-UCC could help alleviate this issue; more research will need to be performed to confirm this.

Compared with standard UCCSD, LAS-UCC can be seen as augmenting UCCSD with a multireference initial state. Instead of using single-determinant Hartree–Fock, as is standard in VQE demonstrations of UCCSD,^58,102−105^ LAS-UCC uses the unentangled product state of the ground-state wave functions of each fragment (which is also the LASSCF wavefunction). This provides additional accuracy, above standard single-reference UCCSD, at a negligible increase in cost. When using a global UCCSD ansatz, the increase in the number of gates is negligible, even when taking into account the *O*(1000) time steps that would be needed to implement the QPE step. Using the *m*-local ansatz provides further reduction. There have been other proposals for preparing interesting, multireference initial states in the context of efficiently finding states with a large overlap with the true ground state.^100,106^ These algorithms could be used in-place of the QPE part of LAS-UCC to provide the initial state and potentially adapted to give similar LASSCF-like states with similar overhead reductions as shown for QPE. We can also provide a qualitative comparison of various other overheads of LAS-UCC with standard UCCSD. A primary overhead with any variational algorithm is the need to optimize many parameters. By selecting only a subset of the cluster operators available, LAS-UCC reduces the total number of parameters and should, therefore, reduce the difficulty of optimization. The initial state preparation becomes more difficult than in standard UCCSD; the standard Hartree–Fock state only takes a depth-1 circuit and happens with probability 1; the LAS-UCC initial state requires one to local-QPE circuits and will need to be executed multiple times, due to the overlap issue in QPE.^91^

Moreover, recent advances in VQE algorithms have developed various ways to reduce the cost associated with the UCC correlator.^45,47,107−110^ As presented in the Theory section, LAS-UCC can also be seen as a post-LASSCF method that recouples select fragments at a level of theory beyond the mean field. The addition of the doubles or higher terms in the cluster operator provides a way to systematically improve the accuracy beyond the LASSCF reference. On classical computers, such an approach requires truncating^68^ or approximating^111^ the non-terminating BCH expansion in a more or less arbitrary way.

Not every system will be accurately described by LAS-UCC, of course, but one can systematically increase the accuracy in several ways, while increasing the total resource cost. Increasing the size of each fragment (which in turn decreases the number of fragments) gradually increases the accuracy, until the limit of a single fragment, where the UCCSD ansatz becomes redundant and the algorithm becomes simply global QPE. On the UCC side, the order of the ansatz can be increased. Triples, quadruples, and so on can be included at increasing cost. If using an *m*-local ansatz, the scaling is unaffected, but the total number of gates increases. The locality of the ansatz, *m*, can also be increased, providing explicit correlation between more geometrically distant fragments. The algorithm can be further improved by using more efficient methods to substitute the QPE or VQE parts of the circuit. For instance, the QPE part of the circuit can be replaced by various asymptotically more efficient time steppers, such as qubitization.^112^ Methods which break the QPE step into multiple circuits, such as iterative phase estimation^113^ or quantum Krylov subspace algorithms^114^ or even by a circuit that efficiently loads the CI vector obtained from LASSCF or methods like DMRG^62^ and Selected CI.^61^ The VQE part can also take advantage of many advances in the field like adaptive derivative-assembled pseudo-Trotter (ADAPT),^107^ qubit CC,^115^ or unitary selective coupled cluster (USCC).^110^ These alternative ansatzes, especially ADAPT and USCC, could potentially automatically generate VQE circuits which naturally have fragmented structures, as those cluster operators will arguably be the most important.

## Conclusions

We introduced LAS-UCC, a quantum algorithm that combines a fragmentation of the wave function of a chemical system with the QPE and variational UCCSD to compute the ground-state energy of such a system. LAS-UCC can describe compounds containing strongly interacting fragments, and it provides a polynomial scaling advantage in the number of quantum gates compared with other quantum algorithms such as QPE and UCCSD. Because the fragments’ reduced Hamiltonians have fewer terms and by ensuring the locality of the Jordan–Wigner transform, the overall gate count will be *O*(*N*) with respect to the total size of the system *N* for linear geometries and *O*(*N*^2^) more generally, compared with *O*(*N*^5^) requirements for QPE. We also demonstrated the accuracy of LAS-UCC on (H_2_)_2_ and *trans*-butadiene molecules and performed resource estimations of larger systems to provide evidence for potential scaling advantages.

As larger fault-tolerant quantum computers are developed, we expect that our algorithm will be able to provide accurate calculations of large and useful chemical systems, such as molecular magnets and qubits, photovoltaic materials, and large biomolecules that are out of reach of classical computing algorithms but for which QPE would be too expensive.

## Methods

### Quantum Algorithms

Here, we describe two quantum algorithms that serve as the primary components for our fragment-based quantum algorithm.

### Quantum Phase Estimation

The QPE algorithm solves for the eigenvalue, λ_*k*_, for an eigenvector  of some unitary matrix, *U*. In addition to its use in quantum chemistry, it forms the basis for many important quantum algorithms, such as Shor’s prime number factoring algorithm^116^ and the Hassidim–Harrow–Lloyd algorithm for inverting matrices.^117^ For quantum chemistry problems, the unitary matrix *U* is generated by the Hamiltonian, *H* (eq 1), over time steps τ
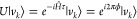
14and the desired energy is mapped to the phase acquired, *E* = −2πϕ/τ, where units have been chosen such that ℏ = 1. By combining real-time evolution of the Hamiltonian, *Ĥ*, with application of the quantum Fourier transform,^118^ the value of the energy can be obtained in polynomial time using a quantum computer.

The computational complexity of the QPE is directly related to the complexity of implementing the unitary propagator . Many strategies for implementing *U* exist, including Trotterization,^119,120^ Taylorization,^121^ and qubitization.^112^ The Hamiltonian, eq 1, has *O*(*N*^4^) terms, where *N* is the number of spin-orbitals. Each term in the Hamiltonian can be transformed into a Pauli string (i.e., a product of Pauli operators *X*, *Y*, *Z*, or *I*) via one of the many fermion-to-spin transformations, such as the Jordan–Wigner,^122^ parity,^123^ and Bravyi–Kitaev^124^ transformations. In this work, we focus on QPE using Trotterization with the Jordan–Wigner transformation because they serve as standard reference points for the other variations. The complexity of QPE for the Hamiltonian, eq 1, using Trotterization with the Jordan–Wigner transformation is *O*(*N*^5^): *N*^4^ arising from the number of terms in the Hamiltonian and an additional *N* from the Jordan–Wigner transform. Although QPE can obtain estimates of the ground-state energy with only a polynomial number of quantum gates, the overheads are still too large for near-term quantum computers. The success of the QPE algorithm directly depends on the overlap of the initial state (which is often taken to be the Hartree–Fock state) and the true ground state. Realistic estimates, taking into account overheads, such as quantum error correction, put the needed number of qubits to perform QPE on interesting molecules in the millions.^95,125,126^

QPE is analogous to a Fourier analysis of a correlation function, and, for a given energy accuracy, ϵ, it requires propagation efforts (maximum times) on the order of *O*(1/ϵ).^47,87^ Because the circuit depth for evaluating the propagator for individual fragments will naturally be lower than for the full system, the QPEs involved in our LAS approach will be significantly cheaper than full QPE.

### Variational Quantum Eigensolver

The VQE is a hybrid quantum-classical algorithm that relies on the variational principle to find an estimate of the ground-state energy of a given molecule. A circuit with variable parameters, θ, serves as an ansatz, whose energy is evaluated on a quantum computer and whose parameters are iteratively optimized by a classical computer. For a circuit ansatz |ψ(θ)⟩, VQE estimates the energy as
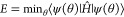
15

The Hamiltonian, *Ĥ*, is transformed into a sum of Pauli strings via a fermion-to-spin transformation, and the expectation value of each term is measured from the quantum computer separately and summed on the classical computer. VQE has much less stringent quantum resource requirements than QPE has because it offloads much of the work (such as optimization) to the classical computer. Hence, VQE has been used in proof-of-principle calculations for small molecules.^102,104,105^

The accuracy of VQE is determined by the quality of the ansatz, |ψ(θ)⟩. The UCCSD ansatz is an interesting choice as a wave function for VQE because there is no known way to efficiently implement UCCSD on classical computers,^127−129^ but it can be implemented with *O*(*N*^5^) gates on quantum computers.^57,130,131^ The UCCSD ansatz is
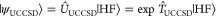
16where  is defined by truncating the more general cluster operator of eq 8 at the second term. While the UCCSD ansatz can be implemented on NISQ devices for small molecules,^58,103^ it is limited in its accuracy because of only including up to double excitations.

### Computational Methods

To calculate the accuracy of the proposed method for small molecules, we use the following strategy. We first use a classical LASSCF solver, as implemented in the *mrh* package,^132^ to find the best product wave function. This effectively provides an equivalent solution to that of the QPE step of our proposed algorithm. We then represent this product wave function as a CI vector in the complete active Fock space and apply a UCCSD correlator, as well as its derivatives with respect to all amplitudes, to this reference CI vector. We employ the factorization reported by Chen et al.^133^ to avoid the BCH expansion and its inevitable approximate truncation.

We note that this factorization corresponds to the “dis-entangled” form of UCC, in which the result is sensitive to the order in which the individual coupled cluster generators are applied to the ket.^102,134,135^ Our choice of operator ordering in this work was arbitrary and based on the ease of implementation. For the LAS-UCC calculations, first singles and then doubles operators were applied, and within each order, generators were sorted first by spatial orbital addressed and then by spin-state addressed. The spatial-orbital strings for singles correlators [i.e., *x*_*l*_^*k*^ from eq 8] span the lower triangular space [*k* > *l*] and are advanced in “row-major order” (e.g., *l* is advanced, and then *k*). For the spatial-orbital index strings of the doubles correlators (*x*_*ln*_^*km*^), the formally nonredundant generators with *k* ≥ *m*, *l* ≥ *n*, and *km* ≤ *ln* are applied in the order that the elements of a 4-dimensional C-language (i.e., “row-major”) array with indices *k*, *m*, *l*, *n* are stored in memory. Spin-orbital strings are sorted as the elements of a C-language array with indices (*m*)*k*, (*n*)*l* for singles (doubles) generators, where spin-up and spin-down, respectively, map to 0 and 1. Spin index strings, which result in changing the total number of spin-up and spin-down electrons, or in formally redundant (e.g., *x*_*kl*_^*mn*^ after *x*_*mn*_^*kl*^) or undefined (e.g., *x*_*kl*_^*kl*^) generators after combining with the spatial-orbital index strings, are omitted. For more detail, see Listings 1–3 in the Supporting Information. For the UCCSD calculations, we use ordering consistent with Qiskit’s UCCSD generation and summarize the ordering in Listing 4 of the Supporting Information.

The resulting |QLAS⟩ CI vector and its derivatives (|δQLAS⟩) with respect to the UCC amplitudes are used to compute the energy, , and its derivatives, . We then minimize the former using the latter and the Broyden–Fletcher–Goldfarb–Shanno algorithm. We find that this approach is more efficient than directly simulating the quantum circuits. We note that this method scales exponentially on classical computers.

To provide gate count estimates, we use the Q# package,^136^ generally following the framework of ref (137). The full and reduced Hamiltonians are produced by using the *mrh* package,^132^ and both Hamiltonians are then passed to the Q# package to estimate the number of CNOT gates using the QPE algorithm with a single Trotter time step for each. Additionally, we estimate the number of CNOT gates necessary to calculate various UCCSD ansatzes, including a global UCCSD ansatz over the whole unfragmented molecule and multiple 2-local ansatzes that span only two fragments. We count only the number of logical quantum gates needed. Real quantum computers will require additional overheads, owing to limited connectivity and the need to use expensive quantum error correction protocols to deal with inevitable errors.^95,126^ Furthermore, we provide gate counts only; no attempt was made to count gate depth, which is typically smaller, because many gates can be implemented in parallel.

## Supporting Information Available

Description of LAS-VQE method with test calculations; discussion of orbital ordering and the Jordan-Wigner transformation; resource estimates of single qubit rotation gates; discussion of strong correlation in the trans-butadiene molecule and potential energy curves for various classical methods; and listings showing operator orderings in LAS-VQE and UCCSD calculations (PDF)
