# Supplementary material for: Localized Quantum Chemistry on Quantum Computers
Source: J Chem Theory Comput. 2022 Nov 8;18(12):7205–17. doi: 10.1021/acs.jctc.2c00388 (PMC9753592; doi:10.1021/acs.jctc.2c00388)
Supplement: Supplementary file 1 — ct2c00388_si_001.pdf [file ct2c00388_si_001.pdf]

Supporting Information: Supplementary Information for Localized Quantum Chemistry on Quantum Computers

Matthew Otten,[*,†] Matthew R. Hermes,[‡] Riddhish Pandharkar,[‡] Yuri Alexeev,[¶] Stephen K. Gray,[*,§] and Laura Gagliardi[*,‖]

†HRL Laboratories, LLC, 3011 Malibu Canyon Road, Malibu, CA 90265

‡Department of Chemistry, Pritzker School of Molecular Engineering, James Franck Institute, Chicago Center for Theoretical Chemistry, University of Chicago, Chicago, IL 60637, USA.

¶Computational Science Division, Argonne National Laboratory, Lemont, IL 60439, USA

§Center for Nanoscale Materials, Argonne National Laboratory, Lemont, IL 60439, USA

‖Department of Chemistry, Pritzker School of Molecular Engineering, James Franck Institute, Chicago Center for Theoretical Chemistry, University of Chicago, Chicago, IL 60637; Argonne National Laboratory, Lemont, IL 60439, USA.

E-mail: mjotten@hrl.com; gray@anl.gov; lgagliardi@uchicago.edu

Contents

LAS-VQE **S-2**

Justification of Eq. (9) of the main text **S-5**

Orbital Ordering and the Jordan-Wigner Transformation **S-6**

Single Qubit Rotation Gates **S-8**

Strong correlation of *trans*-butadiene **S-8**

Classical multireference methods for *trans*-butadiene **S-11**

UCCSD operator ordering in LAS-UCC **S-11**

Geometries and structures **S-18**

References **S-19**

LAS-VQE

Here we describe a more approximate approach than LAS-UCC wherein the QPE circuits for the fragments are replaced by UCC ansatzes, leading to a fully variational method. The chemical knowledge that guides us in defining subspaces of the LAS wave function can also be used to reduce the size of the unitary operator for the UCC ansatzes. It suggests that operators corresponding to the higher excitation from one fragment to another do not affect the wave function significantly. Thus, we introduce a modified ansatz for truncated UCC circuits where we consider the "locality" of the excitation. An excitation involving only orbitals localized on a particular fragment—that is defined by the user—is classified as a "local excitation." In this modified UCC ansatz the user not only can truncate the UCC excitation to a certain maximum number (doubles, triples, etc.) but also can impose a constraint of locality on the higher excitations. We develop here the theory and the algorithm of this method and name it LAS-VQE. In LAS-VQE, all the singles excitations are included while only the local ones are included for the higher excitations. This corresponds to having a mean-field interfragment interaction and allowing all possible orbital rotations. This does not reduce the number of qubits required to represent the system, but it does lower the complexity of the circuit as discussed below. In principle, one could use the locality argument to include only local excitations, allowing us to reduce the number of qubits required. This separate-fragments VQE approach, however, fails to account for any interaction between the fragments. We choose to always include the singles excitations (orbital rotations) across the entire system to account for some interfragment correlation and to make the method less sensitive to the localization schemes

used.

We also choose to perform in the singles excitation among spin-restricted orbitals. We achieve this by using the same parameter to control the alpha and beta excitations corresponding to a given pair of spatial orbitals. This is necessary to avoid artificial lowering of energies due to spontaneous symmetry breaking (the spin unrestricted solution). An example of the effect of this is seen for the hydrogen dimer shown in Figure 2 of the main text. The nonspin adapted UCCS energy is about 0.2 mHartree lower than the Hartree–Fock and has an spin contamination of 0.09, showing the symmetry breaking. Working with spin-restricted orbitals not only allows us to prepare symmetry preserving states for the subsequent excitations, but also lowers the number of parameters at the singles level by a factor of 2. This becomes a significant advantage for the classical optimizer in VQE as the size of the system gets larger. We study the ground state electronic structure of

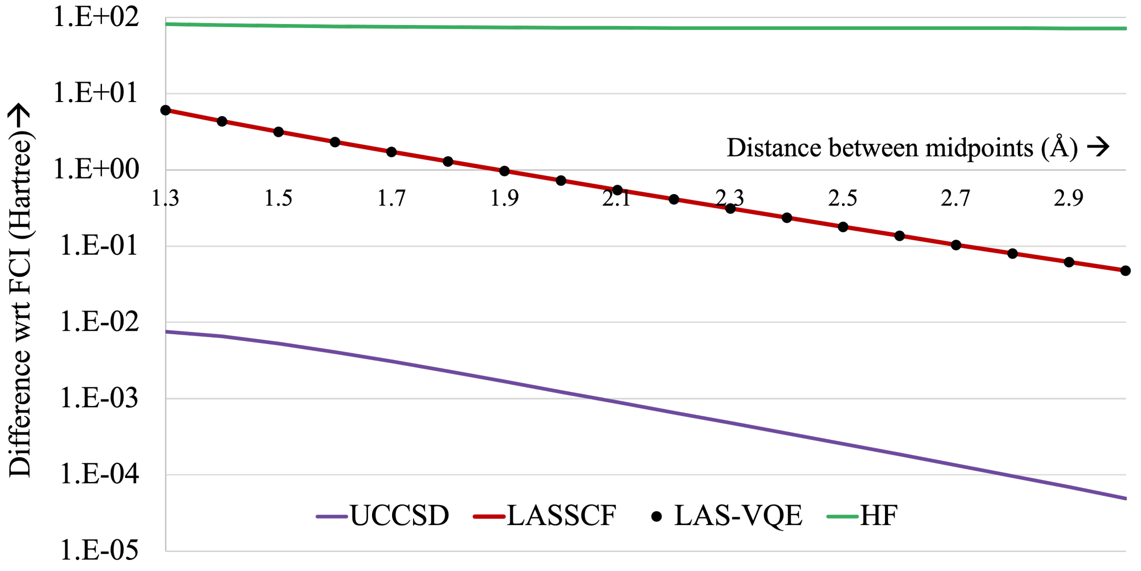

Figure S1: Energy difference (in Hartree) between FCI and the approximate methods as a function of the distance between the midpoints of the two H_2 molecules

the hydrogen dimer in various conformations with the STO-3G basis set. We use CASSCF with a (4,4) active space and LASSCF with a ((2,2),(2,2)) active space, UCCSD and LAS-VQE. Each H_2 molecule is considered as one fragment on which the molecular orbitals are localized. As one would expect, the performance of LASSCF with respect to CASSCF (which is also FCI in

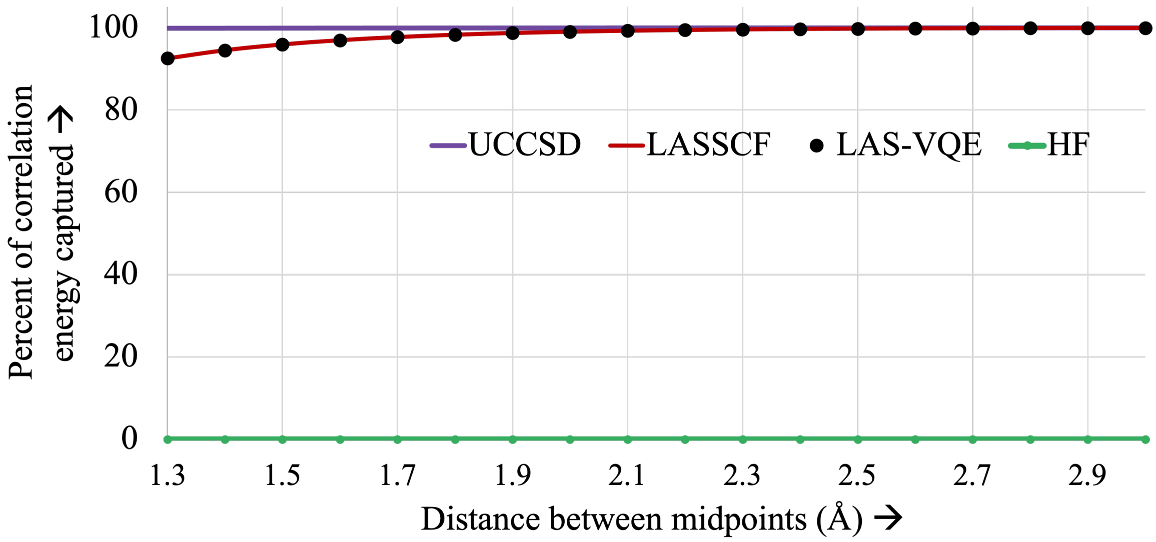

Figure S2: Percentage of correlation energy accounted for by the various methods for different intermolecular distances

this case) worsens with decreasing distance between the two H_2 molecules. Figure S1 shows the energy difference between the various methods and the FCI/CAS(4,4) absolute energies. UCCSD in this case is not exactly FCI but does give accurate results. LAS-VQE is close to (but slightly lower than) the LASSCF energy. The LAS-VQE energy is not variationally bound by the LASSCF that it is based on. In contrast to the LAS-UCC method discussed in this paper, this method uses a single determinant Hartree–Fock reference state. The UCCSD energy is a variational lower bound to the LAS-VQE method. There is, however, a significant advantage when it comes to the computational cost: gate count and the depth of the circuit. The number of doubles operators scales linearly with the number of fragments. The Table S1 shows the number of gates and parameters required for the various calculations for a system with increasing number of H_2 molecules with the same fragmentation scheme. LAS-VQE in considerably lower in both circuit depth and number of parameters, compared with full UCCSD.

Table S1: Circuit depth and number of parameters for the various methods for an increasing number of H_2 fragments

	Circuit Depth			Parameters		
	UCCS	LAS-VQE	UCCSD	UCCS	LAS-VQE	UCCSD
$1 \times H_2$	14	97	97	1	2	2
$2 \times H_2$	58	141	1791	4	6	22
$3 \times H_2$	147	230	11206	9	12	108
$4 \times H_2$	296	379	42789	16	20	344

Justification of Eq. (9) of the main text

Substituting Eqs. (4) and (6) into the Eq. (3) of the main text and differentiating with respect to generator amplitude $x_{\vec{k}}$ yields

$$\frac{\partial E_{\text{LAS}}}{\partial x_{\vec{k}}} = \langle\Phi| \bigwedge_{L\neq K} \langle\Psi_L| \wedge \langle\vec{k}|\hat{H} - E_{\text{LAS}}|\Psi_K\rangle \bigwedge_{M\neq K} |\Psi_M\rangle \wedge |\Phi\rangle\,, \tag{1}$$

the vanishing of which corresponds to the minimization of the LAS energy given by Eq. (3). Within the Hilbert space of the Kth fragment, the energy minimization conditions for the $x_{\vec{k}}$ amplitudes corresponds to an eigenproblem,

$$\hat{H}_K |\Psi_K\rangle = E_{\text{LAS}} |\Psi_K\rangle\,, \tag{2}$$

where

$$\begin{aligned} \hat{H}_K &\equiv \langle\Phi| \bigwedge_{L\neq K} \langle\Psi_L| \hat{H} \bigwedge_{M\neq K} |\Psi_M\rangle \wedge |\Phi\rangle \\ &= \tilde{h}^{k_1}_{k_2}\hat{a}^\dagger_{k_1}\hat{a}_{k_2} + \frac{1}{4}h^{k_1k_3}_{k_2k_4}\hat{a}^\dagger_{k_1}\hat{a}^\dagger_{k_3}\hat{a}_{k_4}\hat{a}_{k_2}, \end{aligned} \tag{3}$$

where $\tilde{h}^{k_1}_{k_2}$ is given by Eq. (10) of the main text. This effective Hamiltonian describes the Kth fragment without interacting with any other fragment; $\hat{H}_K$ for various K mutually commute.

For a system composed of noninteracting fragments A and B with Hamiltonians $\hat{H}_A$ and $\hat{H}_B$,

energies E_A and E_B, and wave functions $|\Psi_A\rangle$ and $|\Psi_B\rangle$, it is generally true that

$$\left(\hat{H}_A + \hat{H}_B\right)|\Psi_A\rangle \wedge |\Psi_B\rangle = (E_A + E_B)\,|\Psi_A\rangle \wedge |\Psi_B\rangle\,. \tag{4}$$

Noting that $\hat{H}_{\text{eff}}$ given by Eq. (9) of the main text is obviously

$$\hat{H}_{\text{eff}} = \sum_K \hat{H}_K, \tag{5}$$

we conclude that

$$\hat{H}_{\text{eff}} \bigwedge_K |\Psi_K\rangle = n_f \times E_{\text{LAS}} \bigwedge_K |\Psi_K\rangle\,. \tag{6}$$

In other words, $\hat{H}_{\text{eff}}$ models interacting fragments as non-interacting subsystems described by unrelated, mutually commuting effective Hamiltonian terms. Thus, the QPE algorithm applied to $\hat{H}_{\text{eff}}$ generates $|\text{QLAS}\rangle \equiv \bigwedge_K |\Psi_K\rangle$ on the quantum circuit.

Orbital Ordering and the Jordan-Wigner Transformation

The Jordan-Wigner transformation[S1] is one of the many ways of transforming fermion operators into spin operators, and is a necessary step for performing quantum chemistry calculations on quantum computers. Here, we very briefly discuss the overheads associated with the Jordan-Wigner transformation and how it can be mitigated in certain fragmented geometries.

The Jordan-Wigner transformation transforms a fermion creation operator for orbital j, $a_j^\dagger$, out of a total of N spin orbitals as follows

$$\tilde{a}_j^\dagger = Z^{\otimes j-1} \otimes \frac{X - iY}{2} \otimes I^{\otimes N-1}, \tag{7}$$

where $\tilde{a}_j^\dagger$ is the transformed operator; X, Y, and Z are the Pauli operators; and the notation $Z^{\otimes N}$ denotes applying the tensor operation, $\otimes$, N times for Z (i.e., $Z^{\otimes 4} = Z \otimes Z \otimes Z \otimes Z$. The term $Z^{\otimes j-1}$ ensures that the transformed operators obey the correct fermionic anti-commutation relations. In

standard quantum algorithms for quantum chemistry, these Z strings introduce very high-weight (that is, having many terms that are not I) terms into the Hamiltonian and cluster operators, leading to an $O(N)$ overhead. To see this, we focus on just the one-body terms. Under the Jordan-Wigner transformation, and assuming $j > k$,

$$\tilde{a}_j^\dagger \tilde{a}_k = I^{\otimes k-1} \otimes \frac{Z(X - iY)}{2} \otimes Z^{\otimes j-k-1} \otimes \frac{X - iY}{2} \otimes I^{\otimes N-j}. \tag{8}$$

The weight (number of non-identity terms) of this operator is $j - k + 1$. In the most general case, there will exist terms where spin orbital 1 and N will have a nonzero component, and the resulting weight is N, which introduces the $O(N)$ overhead normally associated with the Jordan-Wigner transformation. There are many techniques for generally reducing this overhead.[S2,S3] In our simple chain of H_2 molecules, we can remove this scaling by choosing a particular ordering of the orbitals. One typical ordering of the orbitals is the following: all occupied spin up orbitals, all virtual spin up orbitals, all occupied spin down orbital, all virtual spin down orbitals. If this ordering is followed when using the local actives spaces of each fragment, the $O(N)$ overhead is still applicable. Take, for example, just the one-body term from the first occupied spin up orbital of the first fragment to the first virtual spin up orbital for the first fragment. In this case, $j = 1$ and $k = O(N)$, leading to the typical $O(N)$ Jordan-Wigner scaling. If, instead, the orbitals are ordered with all occupied spin up orbitals of fragment one, followed by all virtual spin up orbitals of fragment one, followed the same for the second fragment, and so on, the overhead is removed. Now, $j = 1$ and $k = O(N_k)$, where N_k is the number of spin orbitals in each fragment. Since N_k is assumed to be fixed as the number of fragments grows, the $O(N)$ scaling is reduced to a constant. To remove the Jordan-Wigner overhead from mixed spin two-body terms, the ordering can be further changed so that each spin up orbital is immediately followed by its analogous spin down orbital. This shows that the Jordan-Wigner $O(N)$ overhead can be removed from all terms in the reduced Hamiltonian, removing the $O(N)$ scaling from the QPE part of LAS-UCC. For UCCSD correlators, the Jordan-Wigner overhead can be removed for certain geometries, such as the linear H_2 chain studied in the

main text. More complex geometries, such as higher dimensional lattices, will re-introduce scaling terms, as the orbitals cannot be simultaneously ordered to give low-weight Pauli strings for terms which couple both in the multiple dimensions, such as x and y in a 2D lattice.

Single Qubit Rotation Gates

Figure S3 shows the estimated number of arbitrary single qubit rotation gates needed to implement full QPE across the whole molecule, the fragmented LAS-QPE, full UCCSD, and the 2-local UCCSD correlator with increasing number of H_2 molecules. Just like in the main text, LAS-UCC has polynomially fewer gates, compared with QPE or UCCSD, scaling only linearly for the linear chain geometry. QPE and UCCSD only need $O(N^4)$ single qubit rotations because the Jordan-Wigner overhead only shows up in the CNOT gates.

Strong correlation of *trans*-butadiene

The symmetric double-bond dissociation curve of the *trans*-butadiene molecule (cf. Figs. 2b and 4 of the main text) calculated using the CCSD(T) method is plotted in Fig. S4 and compared to CASCI and LASSCF curves. In the dissociation limit, in which the molecule objectively can be modeled as three non-interacting diradical fragments, the CCSD(T) method recovers a greater amount of correlation energy than LASSCF, *albeit* not as much as LAS-UCC or CASCI. Near the equilibrium geometry, the three methods are indistinguishable.

However, unlike LASSCF, CASCI, or LAS-UCC, all of which are multireference methods, the single-reference CCSD(T) method cannot smoothly connect the region of the equilibrium geometry to the bond dissociation limit. The discontinuous and unphysical behavior of CCSD(T) in the region of $R = 1.8$Å is consistent with the tendency of single-reference coupled cluster at low truncation orders to struggle to dissociate multiple or high-order bonds.[S4] This *trans*-butadiene potential energy curve is an example of a system in which both static and dynamical correlation must simultaneously be accounted for in order to obtain quantitatively accurate results.

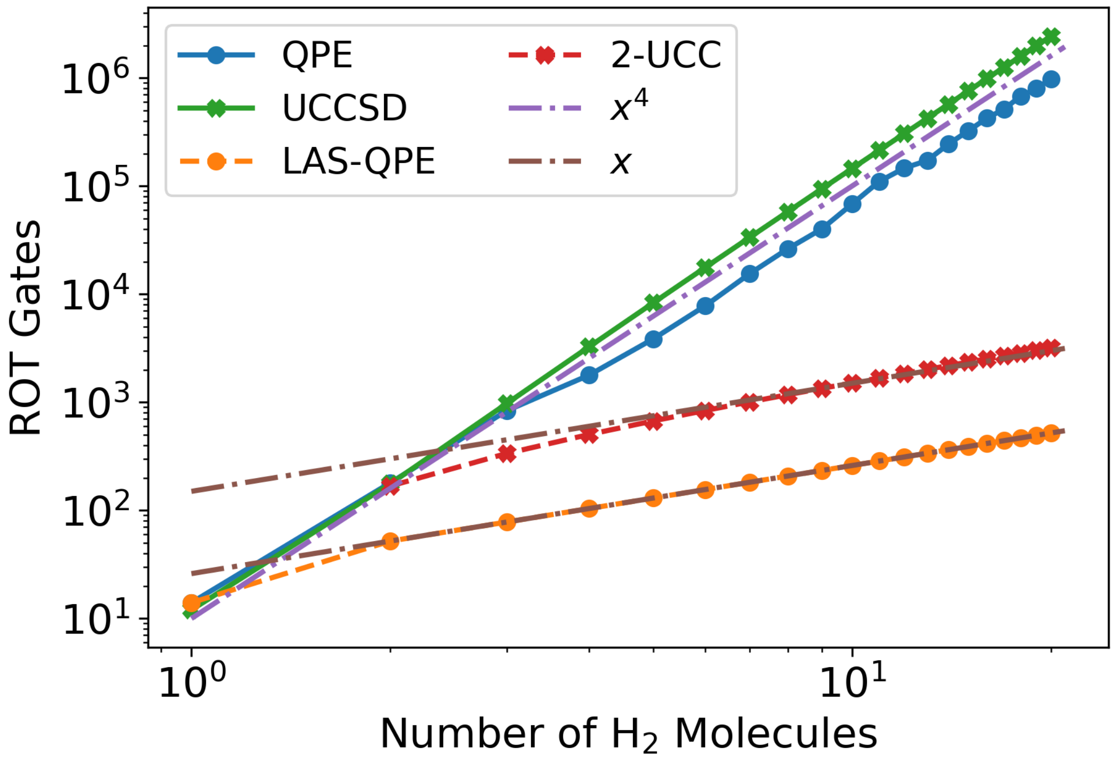

Figure S3: Estimated single qubit arbitrary rotation gate counts using various algorithms. The QPE estimates assume only a single Trotter step. Polynomials of various orders have been plotted to demonstrate the scaling. Our algorithm, LAS-UCC, requires both the LAS-QPE and 2-UCC circuits and thus has an overall $O(N)$ scaling, compared with the $O(N^4)$ scaling of UCC and QPE.

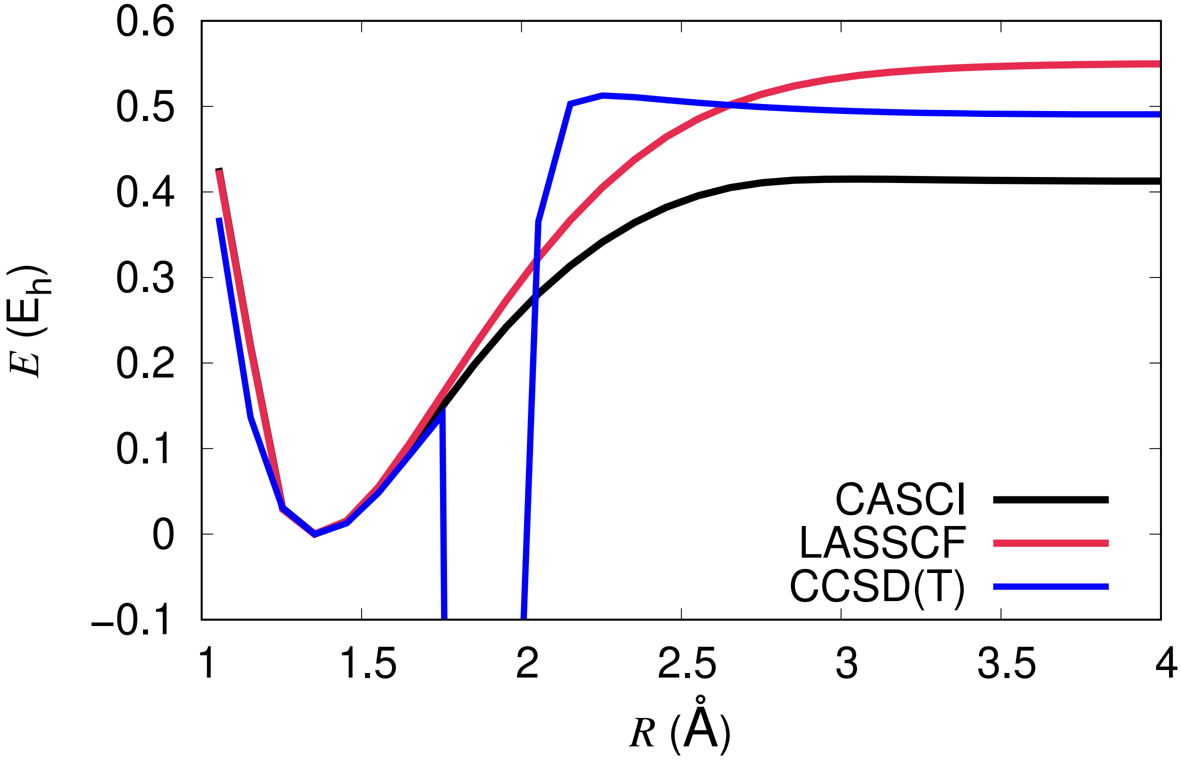

Figure S4: Potential energy curve of the *trans*-butadiene molecule along the symmetric double C=C bond dissociation coordinate, computed with CASCI, LASSCF, and CCSD(T). Total energies for each method are shifted to zero at the equilibrium geometry.

Classical multireference methods for *trans*-butadiene

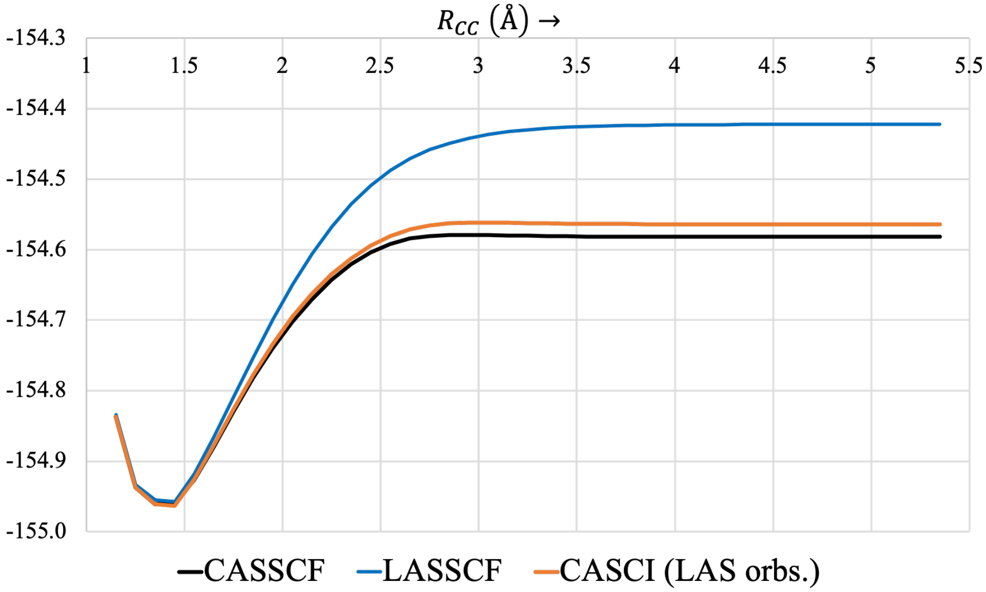

Figure S5: Potential energy curve of the *trans*-butadiene molecule along the symmetric double C=C bond dissociation coordinate, computed with CASCI, LASSCF, and CASSCF

The LASSCF wave function is a variational minimum with respect to all orbital rotations. The converged localized orbitals are used to perform the CASCI calculations. The CASCI and the LASSCF wave function occupy the exact same orbital space but vastly different CI spaces. The CASSCF calculations, however, variationally optimizes the orbitals and has an energy lower than the CASCI wave function. We note that the largest difference between the CASSCF and CASCI energy is 17.7 mHartree while that between CASSCF and LASSCF is 159.6 mHartree. This shows that the dominant source of disagreement between CASSCF and LASSCF is the smaller CI space and not the orbitals space. The active orbitals for the CASSCF and LASSCF calculations of at equilibrium and dissociation geometry are shown in figures S6 and S7.

UCCSD operator ordering in LAS-UCC

The following listings from the *mrh* package describe the operator order for the first-order Trotterized UCCSD correlator used in our classical LAS-UCC calculations. Individual spatial or-

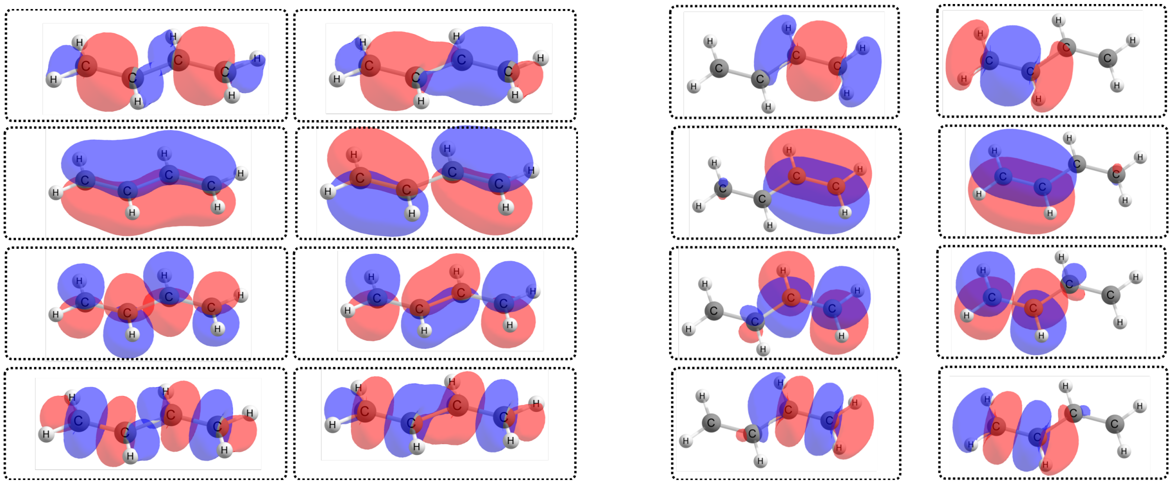

Figure S6: Active orbitals of for CASSCF (left) and LASSCF (right) for the *trans*-butadiene molecule at equilibrium

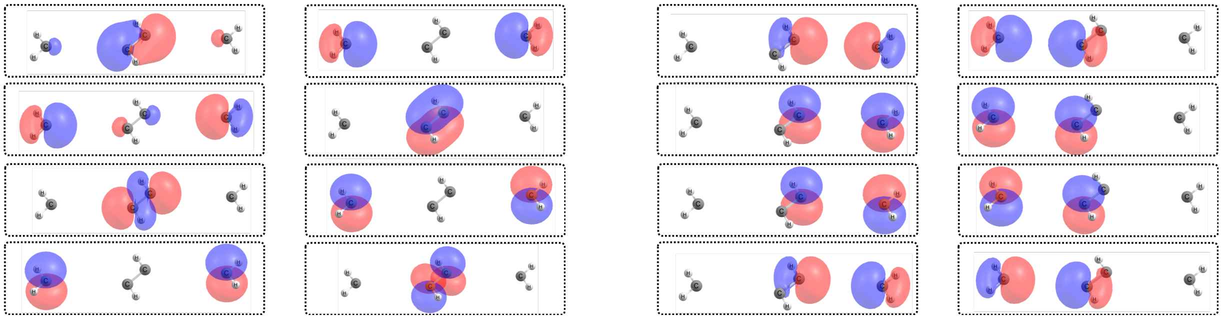

Figure S7: Active orbitals of for CASSCF (left) and LASSCF (right) for the *trans*-butadiene molecule at dissociation

bitals are indexed first by fragment, and then in decreasing order of their natural orbital occupancy (before application of the UCCSD correlator). Listing 1 contains the code for generating the combinations of spatial-orbital excitation patterns, as well as a docstring summarizing the effects of the code presented in Listings 2 and 3 for the case of UCCSD. Listings 2 and 3 contain the code for generating valid spin-orbital excitation patterns for any general set of spatial-orbital excitation patterns of any order in a UCC operator. Note that "np" is numpy and "combinations_with_replacement" is from the itertools module. This code is from the commit identified by the SHA-1 hash 8bb3c2d46ab5f43c8787a365b22713b5410855f2, which differs from the commit which performed the calculations reported in the main text (fe564cd1c54c404edf8bce1dd7118b2d53acdd7a) only in docstrings, test scripts, and line wrapping.

For the UCCSD runs of the main text, Fig. (4), we use ordering consistent with Qiskit's UCCSD generation. We have included a short code listing (Listing 4) which will reproduce the excitation ordering.

Listing 1: Code for generating the spatial-orbital list of the UCCSD correlator in LAS-UCC within *mrh*

```
def get_uccsd_op (norb, t1=None, t2=None):
    ''' Construct and optionally initialize semi-spin-adapted unitary CC
        correlator with singles and doubles spanning a single undifferentiated
        orbital range. Excitations from spatial orbital(s) i(, j) to spatial
        orbital(s) a(, b) are applied to the ket in the order

        U|ket> = u^n(n-1)_nn u^n(n-1)_n(n-1) u^n(n-2)_nn ... u^11_22 u^11_21
                 ... u^n_(n-1) u^n_(n-2) ... u^3_2 u^3_1 u^2_1 |ket>

        where ^ indicates creation operators (a, b; rows) and _ indicates
        annihilation operators (i, j; columns). The doubles amplitudes are
        arbitrarily chosen in the upper-triangular space (a,b <= i,j), but the
        lower-triangular space is used for the individual double pairs
        (a > b, i > j) and for the singles amplitudes (a > i). In all cases,
        row-major ordering is employed.

        The spin cases of a given set of orbitals a, b, i, j are grouped
        together. For singles, spin-up (a) and spin-down (b) amplitudes are
        constrained to be equal and the spin-up operator is on the right (i.e.,
        is applied first). For doubles, the spin case order is

        u|ket> -> ^bb_bb ^ab_ab ^ab_ba ^ba_ab ^ba_ba ^aa_aa |ket>

        For spatial orbital cases in which the same index appears more than
        once, spin cases that correspond to nilpotent (eg., ^pp_qr ^aa_aa),
        undefined (eg., ^pq_pq ^ab_ab), or redundant (eg., ^pq_pq ^ab_ba)
        operators are omitted.

        Args:
            norb : integer
                Total number of spatial orbitals. (0.5 * #spinorbitals)

        Kwargs:
            t1 : ndarray of shape (norb,norb)
                Amplitudes at which to initialize the singles operators
            t2 : None
                NOT IMPLEMENTED. Amplitudes at which to initialize the doubles
                operators

        Returns:
            uop : object of class FSUCCOperator
                The callable UCCSD operator
    '''
    t1_idx = np.tril_indices (norb, k=-1)
    ab_idxs, ij_idxs = list (t1_idx[0]), list (t1_idx[1])
    pq = [(p, q) for p, q in zip (*np.tril_indices (norb))]
    for ab, ij in combinations_with_replacement (pq, 2):
        ab_idxs.append (ab)
        ij_idxs.append (ij)
    uop = FSUCCOperator (norb, ab_idxs, ij_idxs)
    x0 = uop.get_uniq_amps ()
    if t1 is not None: x0[:len (t1_idx[0])] = t1[t1_idx]
    if t2 is not None: raise NotImplementedError ("t2␣initialization")
    uop.set_uniq_amps_(x0)
    return uop
```

Listing 2: Code for generating spin-orbital excitation operators of UCC(SD) in *mrh*. The function "spincases" is presented in Listing 3

```
class FSUCCOperator (uccsd_sym0.FSUCCOperator):
    ''' A callable spin-adapted (Sz only) unrestricted coupled cluster
        operator. For single-excitation operators, spin-up and spin-down
        amplitudes are constrained to be equal. All spin cases for a given
        spatial-orbital excitation pattern (from i_idxs to a_idxs) are grouped
        together and applied to the ket in ascending order of the index

        (a_spin) * nelec + i_spin

        where 'a_spin' and 'i_spin' are the ordinal indices of the spin
        cases returned by the function 'spincases' for a_idxs (creation
        operators) and i_idxs (annihilation operators) respectively, and nelec
        is the order of the generator (1=singles, 2=doubles, etc.) Nilpotent
        or undefined spin cases (i.e., because of spatial-orbital index
        collisions) are omitted.
        '''
    def __init__(self, norb, a_idxs, i_idxs):
        # Up to two equal indices in one generator are allowed
        # However, we still can't have any equal generators
        self.a_idxs = []
        self.i_idxs = []
        self.symtab = []
        for ix, (a, i) in enumerate (zip (a_idxs, i_idxs)):
            a = np.ascontiguousarray (a, dtype=np.uint8)
            i = np.ascontiguousarray (i, dtype=np.uint8)
            errstr = 'a,i={},{}␣invalid␣for␣number-sym␣op'.format (a,i)
            assert (len (a) == len (i)), errstr
            #errstr = 'a,i={},{} degree of freedom undefined'.format (a,i)
            #assert (not (np.all (a == i))), errstr
            if len (a) == 1: # Only case where I know the proper symmetry
                             # relation between amps to ensure S**2
                symrow = [len (self.a_idxs), len (self.i_idxs)+1]
                self.a_idxs.extend ([a, a+norb])
                self.i_idxs.extend ([i, i+norb])
                self.symtab.append (symrow)
            else:
                for ix_ab, (ab, ma) in enumerate (zip (*spincases (a, norb))):
                    if np.amax (np.unique (ab, # nilpotent escape
                        return_counts=True)[1]) > 1: continue
                    for ix_ij, (ij, mi) in enumerate (zip (*spincases (
                            i, norb))):
                        if mi != ma: continue # sz-break escape
                        if np.all (ab==ij): continue # undefined escape
                        if np.all (a==i) and ix_ab>ix_ij:
                            continue # redundant escape
                        if np.amax (np.unique (ij, return_counts=True)[1]) > 1:
                            continue # nilpotent escape
                        self.symtab.append ([len (self.a_idxs)])
                        self.a_idxs.append (ab)
                        self.i_idxs.append (ij)
        self.norb = 2*norb
        self.ngen = len (self.a_idxs)
        assert (len (self.i_idxs) == self.ngen)
        self.uniq_gen_idx = np.array ([x[0] for x in self.symtab])
        self.amps = np.zeros (self.ngen)
        self.assert_sanity ()
```

Listing 3: Code for generating a valid list of spin cases corresponding to a set of field operators for particular spatial orbitals in *mrh*

```
def spincases (p_idxs, norb):
    ''' Compute the spinorbital indices corresponding to all spin cases of a
        set of field operators acting on a specified list of spatial orbitals
        The different spin cases are returned 'column-major order':

        aaa...
        baa...
        aba...
        bba...
        aab...

        The index of a given spincase string ('aba...', etc.) can be computed
        as

        p_spin = int (spincase[::-1].replace ('a','0').replace ('b','1'), 2)

        Args:
            p_idxs : ndarray of shape (nelec,)
                Spatial orbital indices
            norb : integer
                Total number of spatial orbitals

        Returns:
            p_idxs : ndarray of shape (2^nelec, nelec)
                Rows contain different spinorbital cases of the input spatial
                orbitals
            m : ndarray of shape (2^nelec,)
                Number of beta (spin-down) orbitals in each spin case
    '''
    nelec = len (p_idxs)
    p_idxs = p_idxs[None,:]
    m = np.array ([0])
    for ielec in range (nelec):
        q_idxs = p_idxs.copy ()
        q_idxs[:,ielec] += norb
        p_idxs = np.append (p_idxs, q_idxs, axis=0)
        m = np.append (m, m+1)
    p_sorted = np.stack ([np.sort (prow) for prow in p_idxs], axis=0)
    idx_uniq = np.unique (p_sorted, return_index=True, axis=0)[1]
    p_idxs = p_idxs[idx_uniq]
    m = m[idx_uniq]
    return p_idxs, m
```

Listing 4: Code for generating the excitation operators of the UCCSD ansatz for the UCCSD calculations of Fig. (4) of the main text.

```
def generate_fermionic_excitations_occ(num_excitations,num_spin_orbitals,num_particles,
                                       alpha_occ,alpha_unocc,beta_occ,beta_unocc):
    #Assumes ordering [occ_alpha,unocc_alpha,occ_beta,unocc_beta]
    alpha_excitations = []
    # generate alpha-spin orbital indices for occupied and unoccupied ones
    # the Cartesian product of these lists gives all
    # possible single alpha-spin excitations
    alpha_excitations = list(itertools.product(alpha_occ, alpha_unocc))

    # the Cartesian product of these lists gives all
    # possible single beta-spin excitations
    beta_excitations = list(itertools.product(beta_occ, beta_unocc))

    # we can find the actual list of excitations by doing the following:
    #   1. combine the single alpha- and beta-spin excitations
    #   2. find all possible combinations of length `num_excitations`
    pool = itertools.combinations(
        alpha_excitations + beta_excitations, num_excitations
    )

    excitations = list()
    visited_excitations = set()

    for exc in pool:
        # validate an excitation by asserting that all indices are unique:
        #   1. get the frozen set of indices in the excitation
        exc_set = frozenset(itertools.chain.from_iterable(exc))
        #   2. all indices must be unique (size of set equals 2 * num_excitations)
        #   3. and we also don't want to include permuted
        #      variants of identical excitations
        if len(exc_set) == num_excitations * 2 and exc_set not in visited_excitations:
            visited_excitations.add(exc_set)
            occ, unocc = zip(*exc)
            exc_tuple = (occ, unocc)
            excitations.append(exc_tuple)

    return excitations
```

Geometries and structures

H_4 ”close” structure

H 0.0, 0.0, 0.0;

H 1.0, 0.0, 0.0;

H 0.2, 3.9, 0.1;

H 1.159166, 4.1, -0.1

trans-butadiene equilibrium structure

C -1.833376270, 0.000000000, 0.362184378;

H -2.781284200, 0.000000000, -0.139872751;

H -1.857552350, 0.000000000, 1.436642640;

C -0.667087511, 0.000000000, -0.323583629;

H -0.680782100, 0.000000000, -1.400030630;

C 0.667087511, 0.000000000, 0.323583629;

H 0.680782100, 0.000000000, 1.400030630;

C 1.833376270, 0.000000000, -0.362184378;

H 1.857552350, 0.000000000, -1.436642640;

H 2.781284200, 0.000000000, 0.139872751

Table S2: XYZ coordinates for the tris-(μ-hydroxo)-bridged chromium compound

Cr	-1.32078	0.00005	-0.00007
Cr	1.32077	0.00005	-0.00007
O	0.00000	-0.16583	1.45468
O	0.00000	1.34277	-0.58372
O	0.00000	-1.17683	-0.87101
H	0.00002	0.50128	2.15993
H	0.00056	1.61869	-1.51448
H	-0.00044	-2.12079	-0.64413
N	-2.64980	-1.44569	0.71142
H	-2.18696	-2.18198	1.24440
H	-3.05396	-1.84420	-0.13607
H	-3.36727	-1.00512	1.28721
N	-2.64980	1.33902	0.89630
N	-2.64980	0.10677	-1.60777
H	-3.36727	-0.61216	-1.51411
H	-3.05396	0.80432	1.66516
N	2.64980	-1.44568	0.71142
N	2.64979	1.33903	0.89630
N	2.64980	0.10678	-1.60777
H	-2.18697	2.16873	1.26745
H	-3.36727	1.61737	0.22686
H	-2.18696	0.01334	-2.51190
H	-3.05397	1.03998	-1.52914
H	2.18696	-2.18197	1.24440
H	3.05396	-1.84419	-0.13608
H	3.36727	-1.00510	1.28720
H	2.18695	2.16874	1.26745
H	3.05396	0.80433	1.66516
H	3.36726	1.61738	0.22685
H	2.18696	0.01335	-2.51190
H	3.05396	1.03999	-1.52914
H	3.36727	-0.61215	-1.51411

References

(S1) Jordan, P.; Wigner, E. P. *The Collected Works of Eugene Paul Wigner*; Springer, 1993; pp 109–129.

(S2) Tranter, A.; Love, P. J.; Mintert, F.; Coveney, P. V. A comparison of the bravyi–kitaev and

jordan–wigner transformations for the quantum simulation of quantum chemistry. *Journal of Chemical Theory and Computation* **2018**, *14*, 5617–5630.

(S3) Havlíček, V. c. v.; Troyer, M.; Whitfield, J. D. Operator locality in the quantum simulation of fermionic models. *Phys. Rev. A* **2017**, *95*, 032332.

(S4) Li, X.; Paldus, J. Energy versus amplitude corrected coupled-cluster approaches. II. Breaking the triple bond. *J. Chem. Phys.* **2001**, *115*, 5774–5783.
